# The Disinfection of Pulpless Teeth

**Published:** 1896-12

**Authors:** J. W. Wassall

**Affiliations:** Chicago, Ill.


### COMMUNICATIONS.

### The Disinfection of Pulpless Teeth.

BY J. W. WASSALL, M. D., D. D. S., CHICAGO, ILL.

Abstract of paper read before the American Dental Association, Saratoga, N. Y. August, 1896.

Given a tooth with a decomposing pulp, what is the nature of the pathological state with which we have to deal? We find a condition in which the pulp-chamber, root-canals and dental tubules are loaded with putrefactive animal matter. One has but to recall the large amount of soft tissue contained in the dentine and its formative organ to realize the danger of the situation.

Now, while the removal of this necrotic mass from the chamber and canal, and the disinfection and filling of the vacated space, is a manifest necessity, and is the general teaching and practice, I contend that there is not a general appreciation of the fact that the dentine itself continues to remain aseptic.

There are three classes of cases in which we have pollution of dentine by putrefactive products:

1. Teeth, the pulps of which have perished from the encroachment of caries, the pulp-chamber being open.

2. Teeth, the pulps of which have died from proximity to a large filling, attempts at capping, or insufficient sterilization of the layer of caries allowed to remain over a pulp.

3. Pulpless teeth, the canals of which have been imperfectly filled or sterilized, or both.

A tooth, the pulp of which has been devitalized intentionally, is excluded from consideration in this connection for the reason that, under ordinary aseptic precautions, putrefaction does not occur.

The essential pathological condition to be recognized is the same in all three classes given. We have dead dentine infiltrated with matter highly irritating and poisonous to living tissue in intimate contact with vital cementum, which, in turn, is closely enveloped in pericementum.

What must the effect be on the cementum and peridental membrane of the materies morbi which are present in the underlying dentine?

There is no escape from the conclusion that it must account for many morbid conditions and symptoms, the etiology of which is otherwise obscure. There are, no doubt, exceptions of teeth in this state which cause no discomfort. But a little more time may prove that even these cases will not continue quiescent.

Unquestionably, this condition is responsible for numerous affections, more or less difficult of diagnosis, varying from neurosis to remotely situated abscesses, the only subjective symptoms in the causative tooth, discoverable, being a slight sense of lameness in mastication or to palpation.

What treatment does this pathological state indicate? It will not be uninteresting to first notice some of the methods which have, of late, been prominently set forth: The various mummification processes are only temporarily effective because, to use Dr. Harlan's words, "they will not stay mummified."

Emil Schrier destroys pulp-remains with a mixture of sodium and potassium, which is objectionable on the ground that the action of the drug does not extend into the entire depth of the tubules.

Professor Frank Abbott has written a work on "Dental Pathology and Practice" which will be widely read. It is peculiarly unfortunate for the younger members of the profession, whose methods are most likely to be influenced by it, that the chapter, denoted to the consideration of this question, should be so inconsistent with modern bacteriological knowledge. Dr. Abbott imparts a lucid understanding of the fact of putrid dentine and the conditions to be overcome, but the treatment proposed is contrary to the commonest laws of asepsis. The triumph of modern surgery is secured by striving to prevent the entrance of germs into, rather than their destruction after admission to, a wound. How hazardous to teach that a pulpless tooth may be operated upon without first adjusting the rubber dam. The neglect of this precaution against the ingress of the myriads of micro-organisms, which are always present in the human mouth, is hardly excusable at this day. Dr. Abbott also recommends the use of bichlorid solutions for syringing out the pulp-debris. "This is of doubtful utility, because it is at once precipitated in the presence of albumen, thus losing its germicidal and antiseptic powers" (McFarland).

It is also a bad practice to pump zinc chlorid through a tooth to cauterize a pus-sack until a course of treatment for sterilization of the dentine with diffusible disinfectants has first been completed.

These few criticisms are submitted in order to substantiate the charge made at the opening of this paper, that much of the modern practice in the management of pulpless teeth does not conform to the present status of bacteriological science.

The successful treatment of teeth contaminated with putrid pulp-matter would, to my mind, seem to depend upon the strict observance of two details of procedure: First, the exclusive use of diffusible disinfectants.

Second, the repeated and continued application of the disinfectant dressings for a sufficient length of time.

The reason why I give diffusible disinfectants the preference over coagulating drugs is because I am satisfied that exhibition at the pulpal orifices of dentinal tubules forms a plug of coagulated matter which prevents the further ingress of the disinfecting agent, imprisoning within the tubules putrefactive matter which will be a permanent source of irritation to the cementum and pericementum. If it is true, as maintained by Drs. Truman and Kirk, that the entire contents of the tubules is coagulated, there is nothing gained, for the resulting mass is suitable pabulum for micro-organisms, hence there would be no assurance that putrefaction would not recur within the tubules. In the investigations thus far published, the preponderance of evidence seems to be in favor of the avoidance of the coagulating drugs in the roots of pulpless teeth.

The second requisite to success in the disinfection of putrid dentine mentioned, was the element of time: How long shall a dressing be kept in the canal before it is proper to fill it? Until the dentine is permeated throughout its entire depth, and until all micro-organisms and their spores may reasonably be expected to be destroyed.

My observations are that these results are obtained in not less than twelve days—oftener sixteen or twenty—and in some few cases a longer course of treatment is required. The dressings should be changed every four days until no stain or odor is perceived on the cotton-dressing other than the drug employed. Even though the dressing may come away clean on the fourth or eighth day, and you feel certain that the bacteria are killed, it is imperative that the use of the drugs be continued for the full period, in order to also kill any spores which may be present, for they offer greater resistance to germicides, and hence require more time for their destruction.

### DISCUSSION.

Dr. F. Abbott, New York, said that the crowning point of all scientific treatment is the result obtained. You may say there are spores and bacteria, etc., but if they can not develop, what is the difference? You treat these teeth day after day, and what does it amount to? They are plugged up and filled with an antiseptic. This may act as an irritant. He had seen cases, treated in this way, where blood and pus were discharged after three or four days' treatment. He applies an antiseptic and keeps the parts antiseptic by not drying it out, filling in over this. He believes that coagulators should be used in the treatment of pulpless teeth, and that there is no material equal to chlorid of zinc for the purpose. He asserted that if the method, as advocated in his book, was followed, there would not be one failure in a hundred cases so treated.

Dr. Patterson, Kansas City, Mo., said that if you take a tooth that was diseased a considerable time and then filled, in the manner advocated by Dr. Abbott, you will, find by cutting into the dentine, that it was not sterilized. This ought to satisfy any man that his method is an incorrect one, and that the dentine is left in a deplorable condition. The disinfectant should penetrate every zone of dentine as many of the after troubles must result from leaving a zone of dentine in such a deplorable condition as found. He did not propose to take such risks.

Dr. W. H. Morgan, Nashville, Tenn., said that many supposed that the treatment of pulpless teeth was a modern method. In 1847 teeth were treated about as they are to-day. The main difference being that they drilled them out as much as they dared. The canals were then treated with antiseptics, mainly creosote, carried into them on pieces of floss silk, which was well packed into the canal. Their test for thorough disinfection was absence of odor. He could show teeth filled forty years ago by this method that were in good condition to-day.

The teeth are easier to treat successfully in patients of good constitution. The teeth of mulattoes are exceptionally hard to save after the pulp has become devitalized; they seem to take on inflammation and cause trouble. In fifty years of practice he had seen only two mulattoes, adults, who had perfectly sound teeth. He thought intermarriage to blame for these conditions.

Dr. Barrett, Buffalo, said the contents of the tubuli is albuminoid in character and, practically, organized tissue. It will coagulate spontaneously if given a chance. It will melt out, for this is one of the laws of disintegration. It is not the character of the medicament that determines his choice of the disinfectant, for he believes it impossible to get anything into the tubules that will pass through to the pericementum. If we seal the mouth of the tubules it is not necessary to go farther, for the amount of material left in the tubules is so small that no harm will come from it. He thinks that we are getting into speculative philosophy a little too deep. The middle ground between the utter want of scientific treatment, as exhibited by Abbott, and the highly scientific treatment proposed by the essayist, was the best to take. He believes that every dentist should do his best. That honesty is the normal condition of man, and until our instruction has been bad we will remain honest and choose what is best.

Dr. Rhein, New York, believes that while coagulants are not the barriers that is claimed, he does believe that they make a certain barrier to the diffusion of medicaments. By acidulating solutions of bichlorid of mercury you can do away with the coagulating effects. He has found no method so effective in the treatment of pulpless teeth as that advocated by Dr. Schrier. The tubuli are left more open than by any other method he has used and, consequently, the essential oils are more readily diffused after this treatment. He has observed cases thus treated where the taste of the oily dressing came through the cementum after forty-eight hours from the time applied.

Dr. Wassall, Chicago, in closing the discussion, said that the most perfect method is the one to adopt—that sublimate precipitates in the presence of albumen and becomes inert. In using the combination of bichloride and peroxide of hydrogen the results come, probably, from the peroxide, as the bichloride is precipitated. The discoloration of the cotton used for the dressing in a root-canal is not from the drug, but from the putrid matter in the tubules that exudes by osmosis.

